# Safety Profile of Biologics Used in Rheumatology: An Italian Prospective Pharmacovigilance Study

**DOI:** 10.3390/jcm9041227

**Published:** 2020-04-24

**Authors:** Maria Antonietta Barbieri, Giuseppe Cicala, Paola Maria Cutroneo, Elisabetta Gerratana, Caterina Palleria, Caterina De Sarro, Ada Vero, Luigi Iannone, Antonia Manti, Emilio Russo, Giovambattista De Sarro, Fabiola Atzeni, Edoardo Spina

**Affiliations:** 1 Department of Clinical and Experimental Medicine, University of Messina, 98125 Messina, Italy; mbarbieri@unime.it (M.A.B.); gcicala@unime.it (G.C.); elisabettagerratana@gmail.com (E.G.); fatzeni@unime.it (F.A.); 2 Sicilian Regional Pharmacovigilance Centre, University Hospital of Messina, 98125 Messina, Italy; pmcutroneo@unime.it; 3 Department of Health Science, School of Medicine, University of Catanzaro, Clinical Pharmacology and Pharmacovigilance Unit, Mater Domini Hospital, 88100 Catanzaro, Italy; palleria@unicz.it (C.P.); catedesarro@gmail.com (C.D.S.); adavero@hotmail.it (A.V.); iannone@unicz.it (L.I.); mantiantonia7@gmail.com (A.M.); erusso@unicz.it (E.R.); desarro@unicz.it (G.D.S.)

**Keywords:** pharmacovigilance, biologic drugs, inflammatory arthritis, adverse events, real-world data, treatment failure

## Abstract

Post-marketing surveillance activities are essential to detect the risk/benefit profile of biologic disease-modifying antirheumatic drugs (bDMARDs) in inflammatory arthritis. The aim of this study was to evaluate adverse events (AEs) in patients treated with bDMARDs in rheumatology during a prospective pharmacovigilance study from 2016 to 2018. Descriptive statistical analyses were performed to evaluate bDMARDs-related variables of patients without AEs/failures vs patients with AEs and failures. The risk profile among biologics was assessed by comparing patients treated with each bDMARD to patients treated with etanercept. A total of 1155 patients were enrolled, mostly affected by rheumatoid arthritis (46.0%). AEs and failures were experienced by 8.7% and 23.3%, respectively. The number of comorbidities significantly influenced the onset of AEs, while anxiety-depressive, gastrointestinal disease, and fibromyalgia influenced onset of failures. The probability of developing an AE was significantly lower in patients treated with secukinumab, while the probability of developing treatment failure was significantly lower in patients treated with golimumab, secukinumab and tocilizumab. A total of 216 AEs were reported (25.5% serious), mostly regarding infections (21.8%), musculoskeletal (17.6%) and skin (16.2%) disorders. Serious AEs included neutropenia (12.7%), lymphocytosis (9.1%) and uveitis (7.3%). The obtained results revealed known AEs but real-world data should be endorsed for undetected safety concerns.

## 1. Introduction

Inflammatory arthritis refers to a group of chronic systemic disorders including rheumatoid arthritis (RA), psoriatic arthritis (PsA), ankylosing spondylitis (AS), and non-radiographic axial spondyloarthritis (nr-AxSpA) which have a significant negative impact on patient quality of life. These diseases are psychologically and physically debilitating and lead to progressive functional limitations and often irreversible disabilities [1,2,3]. The prevalence of these disorders changes among different patient populations depending on genetic and environmental factors [4]. RA is considered the most frequent chronic rheumatic disease with a prevalence estimate of 0.24% worldwide and 0.33–0.48% in Italy [5,6]. The global prevalence of AS ranges between 0.02–0.35%, while the prevalence of PsA is 0.01–0.19% [7]; in Italy, AS and PsA have a prevalence of 0.37% and 0.42%, respectively [8]. Concerning physiopathology, a combination of genetic, immunological, microbial, and environmental factors, with several genes and signaling immune pathways involved in each disease, is the basis of the pathogenesis of chronic inflammation [9,10].

Regarding the treatment of inflammatory arthritis, conventional synthetic disease-modifying antirheumatic drugs (csDMARDs), including sulfasalazine (SSZ), leflunomide (LFN), hydroxychloroquine (HCQ), and methotrexate (MTX), have been used for more than 50 years in association with corticosteroids (CCs) and non-steroidal anti-inflammatory drugs (NSAIDs) [11]. Today, the introduction of several biologic disease-modifying antirheumatic drugs (bDMARDs) and their biosimilars represents a turning point in treatment. Five different bDMARDs acting on tumor necrosis factor α (TNFα) are available in Italy: etanercept (ETN), infliximab (IFX), adalimumab (ADA), certolizumab (CZP), and golimumab (GOL). Biologics acting on other targets have followed the introduction of TNF-inhibitors (TNFi): abatacept (ABT), a T cells co-stimulation inhibitor; secukinumab (SEC), an IL-17 inhibitor; Anakinra (ANA), an IL-1 inhibitor; rituximab (RTX), an anti-CD20 drug; tocilizumab (TCZ) and sarilumab (SAR), both IL-6 receptor inhibitors; ustekinumab (UST), an anti-IL-12/23. In addition, a low number of targeted synthetic disease-modifying antirheumatic drugs (tsDMARDs) have recently been identified: phosphodiesterase 4-inhibitor apremilast and Janus kinase (JAK) inhibitors, tofacitinib, baricitinib, and upadacitinib [12,13].

Prescriptions for bDMARDs rise with increasing duration of disease and TNFi are the most frequently prescribed drugs, in particular ETN and ADA [14,15,16,17]. Since the rapid development of bDMARDs, the risk/benefit profile of these therapies has still not been completely defined, specifically for long-term treatments. Current limited knowledge on the safety profile of new bDMARDs is the result of randomized controlled trials (RCTs) based on short follow-up periods usually of weeks or a few months and on strict inclusion and highly selective criteria of participants, which thus does not allow detection of all adverse events (AEs) [18,19]. Moreover, it may be necessary to widen information available from post-marketing studies especially for missing data (e.g., effectiveness, subgroups of high-risk patients, interactions, and predictive factors). bDMARDs are associated with AEs related to their specific mechanisms of action but they can also trigger unwanted immune responses (immunogenicity) with the production of anti-drug antibodies [20,21]. Additionally, the immune downregulation associated with these molecules could increase the risk of malignancies [22]. To date, reactivations of infections (e.g., tuberculosis and hepatitis B virus) with TNFi and opportunistic infections [23,24,25], cardiovascular risk, including acute myocardial ischemia [26,27], neurological events [28], and cancer [29], have been observed in patients treated with bDMARDs. Furthermore, these drugs could be the cause of serious AEs (SAEs) and rare and unpredictable AEs, difficult to detect in pre-marketing clinical trials. Post-marketing surveillance activities have played a major role in significantly improving the detection and reporting of SAEs and unexpected AEs in a real-world setting, providing important safety data for several treatments.

The aim of this study was to evaluate the occurrence of AEs and therapeutic failures associated with bDMARDs used in rheumatology units during a prospective pharmacovigilance study in Southern Italy.

## 2. Materials and Methods

### 2.1. Study Design and Data Collection

A multiregional active prospective pharmacovigilance study was conducted between 1 January 2016 and 31 December 2018, for the evaluation of the safety of biologics in nine clinical rheumatology units in the Calabria and Sicily regions: Rheumatology Outpatient Clinic, Azienda Ospedaliera “Pugliese-Ciaccio”, Catanzaro, Italy; Rheumatology Unit, Grande Ospedale Metropolitano “Bianchi-Melacrino-Morelli”, Reggio Calabria, Italy; Rheumatology Outpatient Clinic, Azienda Ospedaliera “Mater Domini”, Catanzaro, Italy; Rheumatology Outpatient Clinic, Azienda Ospedaliera Provinciale Crotone, Italy; Rheumatology Unit, Azienda Ospedaliera “SS Annunziata”, Cosenza, Italy; Rheumatology Outpatient Clinic Azienda Ospedaliera Cosenza, Italy; Rheumatology Unit, Ospedale Castrovillari, Italy; Rheumatology Outpatient Clinic, and Azienda Sanitaria Provinciale Vibo Valentia, Italy; Rheumatology Unit, University Hospital of Messina, Italy. A monitor, a specialist in clinical pharmacology and who received specific training of pharmacovigilance, was assigned for each rheumatology ward.

All patients aged 16 or older and in treatment with a bDMARD for inflammatory arthritis at the time of study beginning (index date) were enrolled and followed for a maximum of three years. All bDMARD-naïve patients during the study period were also included. All the following information was collected: age, sex, clinical diagnosis (i.e., RA, PsA, SA, nr-AxSpA), disease duration, smoking, comorbidities coded by the International Statistical Classification of Diseases and Related Health Problems 10th Revision (ICD-10) and assessed by Charlson score [30,31], concomitant csDMARDs and CCS therapies, current bDMARD treatment, cause of discontinuation and switch/swap to another drug, potential primary or secondary failures and the possible onset of AEs.

Patients were considered to have discontinued treatment if biologics had not been taken within the recommended time or if the therapeutic plan had expired and was not renewed. Furthermore, patients were classified as switchers if they were on treatment with a different bDMARD during the study follow-up period compared to the one used at index date. 

Clinical pharmacologists supported physicians in identifying primary or secondary failures and potential AEs, through an accurate analysis of medical records. For each AE observed, the physician filled in the suspected adverse drug reaction (ADR) reporting form of the Italian Medicines Agency (Agenzia Italiana del Farmaco, AIFA) recording a detailed description, including time to onset and recovery, seriousness, and outcome, codifying the AE according to the Medical Dictionary for Regulatory Activities (MedDRA^®^, The International Federation of Pharmaceutical Manufactures & Associations, IFPMA, Geneva, Switzerland) Preferred Term (PT) and System Organ Class (SOC) levels. An AE was defined as serious if it was life-threatening or fatal, required hospitalization (or prolonged existing hospitalization), resulted in persistent or significant disability or in a congenital anomaly/birth defect, or was any other medically important condition [32]. 

A patient encrypted code was used to maintain anonymity. Collected data were entered into a database developed ad hoc and all patients were classified into three different groups: patients without AEs/failures, patients that developed at least one AE (patients with AEs), and those with a primary/secondary failure (patients with failures).

Informed consent was obtained from all patients at the time of enrolment. All procedures were executed in accordance with the 1964 Declaration of Helsinki and its later amendments. The study protocol was approved by the local Ethics Committees of Calabria Region (protocol number 278/2015) and Messina University Hospital (protocol number 125/2016).

### 2.2. Data Analysis

For each of the three groups defined above, descriptive statistical analyses were performed to evaluate clinical and demographic characteristics of enrolled patients at index date and bDMARD-related variables of patients without AEs or primary/secondary failures compared to patients with AEs and with primary/secondary failures. In addition, information on bDMARD discontinuation and switches/swaps was evaluated. Medians with interquartile ranges (Q1–Q3) for continuous variables and absolute and percentage frequencies for categorical variables were estimated. A non-parametric approach was applied for all variables as some of the numerical variables were not normally distributed according to the Kolmogorov–Smirnov test. In detail, the Mann–Whitney U test for continuous variables and the two-tailed Pearson chi-squared test for categorical variables were used to compare characteristics. A *p*-value ≤ 0.05 was set up as statistically significant.

The risk profile of the onset of AEs or primary/secondary failures among biologics was assessed by comparing patients treated with each bDMARD to subjects in treatment with ETN taking into account that ETN was the first drug authorized for RA and was the most widely used drug in our sample. Crude odds ratios (ORs) and 95% confidence intervals (CIs) were calculated through a univariate logistic regression model. To confirm previous results, a multivariate logistic regression model (backward procedure, α = 5%) was performed adjusting the ORs for those variables that were significant in the descriptive analyses after careful clinical evaluation of possible correlations between variables by applying Spearman’s rank correlation coefficient. Furthermore, an evaluation of AEs and the respective seriousness for each bDMARD was assessed. Statistical analysis was conducted with SPSS version 23.0 (IBM Corp. SPSS Statistics, Armonk, NY, USA). 

## 3. Results

### 3.1. Study Population

A total of 1155 patients were enrolled during the study period: 490 (42.4%) were from the region of Sicily and 665 (57.6%) from Calabria. Patients were followed-up for a median of 23 (22–35) months and were mainly women (*n* = 753; 65.2%) with a median age (Q1–Q3) of 57.0 (48.0–65.0) years and most affected by RA (*n* = 531; 46.0%) followed by PsA (*n* = 442; 38.3%), AS (*n* = 164; 14.2%), and nr-AxSpA (*n* = 18; 1.6%) with a median age (Q1–Q3) of disease duration of 8.0 (4.0–12.0) years. The median age (Q1–Q3) of patients at diagnosis was 48.0 (39.0–56.0) years. More than 40% of patients had at least one comorbidity: hypertension (*n* = 228; 19.7%), disorders of the thyroid gland (*n* = 102; 8.8%), dyslipidemia (*n* = 79; 6.8%), and fibromyalgia (*n* = 74; 6.4%) were the most frequently reported. At index date, more than 50% of patients were in treatment with ETN or ADA (*n* = 342; 29.6% and *n* = 261; 22.6%, respectively). ETN was mostly used in patients with PsA (*n* = 157; 35.5%) and RA (*n* = 136; 25.6%) while ADA in patients with AS (*n* = 46; 28.0%) and nr-AxSpA (*n* = 7; 38.9%). IFX (*n* = 107; 9.3%), TCZ (*n* = 100; 8.7%), ABT (*n* = 95; 8.2%), GOL (*n* = 91; 7.9%), SEC (*n* = 78; 6.8%), UST (*n* = 35; 3.0%), CZP (*n* = 31; 2.7%), RTX (*n* = 11; 1.0%), ANA (*n* = 2; 0.2%), and SAR (*n* = 2; 0.2%) were the other prescribed drugs. 

Only 401 patients (34.7%) were bDMARD-naïve. Median age (Q1–Q3) at biologic index date was 53.0 (44.0–60.0) years. Regarding patients non-bDMARD-naïve, median (Q1–Q3) duration of biologic therapy at index date was 4.0 (3.0–7.0) years. Overall, 480 patients (41.6%) received at least one concomitant csDMARDs and/or CCS therapies, and MTX (*n* = 384; 33.2%) was the most commonly used. 

### 3.2. Safety Profile and Treatment Failures

During the three-year period, 785 patients (68.0%) did not develop therapeutic failures or AEs, while 101 patients (8.7%) experienced at least one AE and 269 (23.3%) had at least a primary/secondary failure. No statistical difference was observed in terms of the frequency of AEs between naïve and previously biologically exposed patients (*n* = 27; 6.7% vs *n* = 74; 9.8%, *p* = 0.098); however, bDMARD-naïve patients experienced a therapeutic failure more frequently compared with those that were already in treatment with a bDMARD (*n* = 111; 27.7% vs *n* = 158; 21.0%, respectively, *p* = 0.012). Table 1 summarizes the main differences of the three groups described above. Females were significantly associated with the onset of AEs and primary/secondary failures. No statistical difference was noticed in terms of age at index date, age at diagnosis, and age at biologic index date among groups. Patients with a diagnosis of RA experienced a therapeutic failure more frequently. The number of comorbidities mainly influenced the onset of AEs. Specifically, disorders of the thyroid gland, osteoporosis, respiratory disease, mixed anxiety-depressive disorder, eye disease, gastrointestinal disease, and uveitis were more significantly identified in this group of patients. Conversely, only mixed anxiety-depressive disorder and gastrointestinal disease, in addition to fibromyalgia, were significantly related to the onset of a treatment failure. Moreover, co-treatment with non-biologics especially cyclosporine, LFN or CCS more likely affected a primary/secondary failure. 

Regarding safety risk profile and treatment failures of bDMARDs compared to ETN, the probability of developing an AE was significantly lower in patients treated with SEC, while the probability of experiencing treatment failure was significantly lower in patients treated with GOL, SEC and TCZ compared to ETN (Table 2). 

Onset of AE was frequently observed among patients with a diagnosis of nr-AxSpA and AS (*n* = 3; 16.7% and *n* = 20; 12.2%, respectively) followed by patients with RA (*n* = 48; 9.0%) and PsA (*n* = 30; 6.8%). A total of 36 patients (35.6%) developed at least one SAE mostly observed in patients with a diagnosis of AS (*n* = 6; 3.6%), followed by RA (*n* = 17; 3.2%) and PsA (*n* = 13; 2.9%). No statistically significant difference was observed for the onset of SAEs among bDMARDs. 

### 3.3. Switches/Swaps and Treatment Discontinuations

Overall, 33 patients (2.9%) discontinued treatment (in detail, 17 patients started a tsDMARDs therapy) and 32 patients (2.8%) were lost to follow-up. Regarding switches/swaps to other bDMARDs, 262 patients (22.7%) started treatments with a different biologic compared to the one used at the index date: specifically, 230 patients (19.9%) were switchers for a primary/secondary failure and only 42 (3.6%) for the onset of an AE. The switch ETN/ADA (*n* = 30; 13.0%), followed by the switch ADA/ETN (*n* = 17; 7.4%) and the swap ABT/TCZ (*n* = 13; 5.7%) were the most frequently performed in patients with a primary/secondary failure, while ETN/ADA (*n* = 6; 14.3%), IFX/ADA, and TCZ/ABT (both *n =* 4; 9.5%) took place in patients with an AE (Table 3). Moreover, 61 patients (23.3%), of whom only 5 (8.2%) for the occurrence of an AE, had at least a second switch/swap to another bDMARD mostly regarding ABT/TCZ (*n* = 10; 16.4%) and ETN/SEC (*n* = 5; 8.2%). Nevertheless, 17 (27.9%) of them reported a third switch/swap, all for therapeutic failure (except for one patient) and mostly regarding GOL/TCZ (*n* = 3; 17.6%). Only two patients had a fourth switch/swap both for a lack of efficacy (i.e., ABT/TCZ and UST/SEC).

### 3.4. Characteristics of Adverse Events

During the study period, a total of 216 AEs were reported in 101 patients that experienced at least one AE (mean of 2.1 AEs per patient). Specifically, 55 (25.5%) were SAEs occurring in 36 patients (mean of 1.5 SAEs per patient). 

According to MedDRA^®^ SOC classification, the most frequently identified AEs were infections and infestations (*n* = 47; 21.8%), musculoskeletal and connective tissue disorders (*n* = 38; 17.6%), skin and subcutaneous tissue disorders (*n* = 35; 16.2%), and general disorders and administration site conditions (*n* = 24; 11.1%). Infections were significantly related to patients treated with GOL (*n* = 10; 11.0%) and ABT (*n* = 9; 9.5%), investigations and nervous system disorders with ADA (*n* = 7; 2.7% and *n* = 5, 1.9%, respectively), musculoskeletal and hepatobiliary disorders with UST (*n* = 6, 17.1% and *n* = 2, 5.7%, respectively), skin disorders with IFX (*n* = 8; 7.5%), general and administration site conditions and blood and lymphatic system disorders with TCZ (*n* = 5; 5.0% and *n* = 4; 4.0%, respectively), eye disorders with ETN (*n*= 5; 1.5%), while respiratory, thoracic and mediastinal disorders with IFX and TCZ (*n* = 4; 3.7% and *n* = 4; 4.0%, respectively) (Table 4).

All AEs belonging to the SOC blood and lymphatic disorders and neoplasm benign, malignant and unspecified were serious (*n* = 13/13; 100% and *n* =2/2; 100%, respectively) (Table 5).

As far as PT classification is concerned, arthritis, intended as exacerbation of arthritic symptoms, was the most frequently reported AE (*n* = 23; 10.6%), followed by a rash (*n* = 10; 4.6%), influenza (*n* = 9; 4.2%), bronchitis, and neutropenia (both *n* = 7; 3.2%). Exacerbation of arthritis and rash were mostly reported among patients treated with UST (*n* = 3; 8.6%, and *n* = 1; 2.9%, respectively), while influenza was reported among patients in treatment with ABT (*n* = 3; 3.2%) and bronchitis in subjects treated with GOL (*n* = 3; 3.3%) and CZP (*n* = 1; 3.2%) (see Table S1). SAEs included seven cases of neutropenia and five cases of lymphocytosis both related to ADA, ETN, TCZ, and IFX; four cases of uveitis mostly observed with ETN and three cases of tooth abscess occurred with ADA and SEC. Furthermore, a case of progressive multifocal leukoencephalopathy (PML), a case of myocardial infarction and a case of breast cancer developed in patients treated with ADA, while a case of basal cell carcinoma was associated to GOL injection. Moreover, ABT was associated with the onset of serious bronchitis, measles and rhinitis but also with the occurrence of a case of chronic obstructive pulmonary disease, a case of skin exfoliation, and an umbilical hemorrhage (Table 5). 

## 4. Discussion

This multiregional study aiming to evaluate the safety profile of bDMARDs in inflammatory arthritis increases awareness of AEs in a real-world setting in the light of the information shown in previous studies [33,34,35,36]. 

Our results reveal a prevalence of females affected by rheumatologic diseases in accordance with the literature [37,38]. Similarly, the median age of patients is consistent with what was observed in previous data [39]. Concerning comorbidities, our findings reported a low prevalence compared to other studies [34,35,40], although one study reported an even lower prevalence (40.9% vs 22.0%) [41]. In addition, our data are consistent with an Italian pharmacovigilance study and an observational, cross-sectional, multicentric study (40.9% vs 40.4% and vs 40.3%, respectively) [18,42]. Hypertension and dyslipidemia were the most common comorbidities [43,44,45]. This could be explained by several mechanisms including genetic polymorphisms and the involvement of immune system activation in the pathogenesis of hypertension [46,47]. Regarding dyslipidemia, the assessment of lipid profiles leads to the ‘lipid paradox’ in patients due to the inflammation process that influences the onset of RA [48]. Thyroid gland disorders, mainly autoimmune hypothyroidism, could be related to inflammatory arthritis with a gap in prevalence for different laboratory hormones cut-offs and various dietary iodine intake levels [49,50]. Hypertension, thyroid dysfunctions, and dyslipidemia are considered risk factors for cardiovascular disease in patients with inflammatory joint disorders [51,52]. Moreover, fibromyalgia is a frequent comorbidity and it may amplify the disease activity score (DAS) and influence the management of patients with rheumatic disease [53]. Regarding bDMARDs, our findings reflect current clinical practice in the use of biologics in the rheumatologic area. In particular, ETN and ADA were the most commonly prescribed drugs in our patients, in accordance with the literature [54,55].

Our data showed a higher significant onset of AEs and failures in females compared to males as previously observed [18,56,57,58]. It is widely known that women have a 1.5–1.7-fold higher risk of AE occurrence compared with men due to differences in terms of pharmacokinetic, immunological and hormonal features [59]. Furthermore, the number of comorbidities was significantly related to the onset of AEs probably for the use of several drugs that predisposes patients to AEs [60]. Conversely, fibromyalgia was significantly associated with primary/secondary failures as it appears to have a negative impact on bDMARDs’ effectiveness in patients with inflammatory arthritis [61,62,63]. Concerning concomitant therapies, a relation between ciclosporin, LFN or CCS and the occurrence of therapeutic failure was noticed. In previous studies, co-treatment with bDMARDs and LFN was related to a higher DAS and discontinuation rate compared to bDMARDs plus MTX; similarly, the co-administration of prednisolone was a negative predictive factor of clinical response and remission in RA [64,65].

Our results reveal a lower probability of experiencing AEs in patients treated with SEC compared to ETN and a lower probability of developing therapeutic failure when treated with GOL, SEC and TCZ compared to ETN. No statistically significant difference was observed for the onset of SAEs among bDMARDs. A switch/swap was observed in slightly more than 20% of patients. As previously shown, in our analysis cessation of an initial bDMARD therapy was related to primary/secondary failure or to AEs [66]. The frequency of switches ETN/ADA, ADA/ETN, and IFX/ADA was comparable with other studies [67,68,69]. 

Considering AEs, our findings are mostly consistent with existing literature and with the Summaries of Product Characteristics (SPCs) available at the time of the study. A higher prevalence of infections, musculoskeletal, skin, and general disorders has been identified. Immunodeficiency, caused by the immunosuppressive effect of bDMARDs, was associated with a higher risk of infections in patients with inflammatory arthritis [70]. Infections were mostly reported in patients treated with GOL and ABT. In contrast with our data, ABT seemed to have a favorable safety profile in comparison with anti-TNF therapies and demonstrated a lower rate of serious infections compared to other bDMARDs, as well as GOL [71,72,73]. The higher risk of infections with ABT in our results could be attributed to use in patients who have known or unknown interstitial lung disease diagnosable only by CT scan [74,75]. Moreover, in other studies, infections were the second reported AEs for GOL and the most frequent AE for ABT [76,77]. Regarding skin disorders, it is well known that anti-TNFα drugs play a role in the onset of dermatological manifestations, including urticaria, erythema and dermatitis, with a frequency ranging between 10–60% [78,79]. In our cases, IFX was more associated with skin reactions and could be considered as one of the risk factors that could lead to cutaneous AEs [80]. Moreover, the SOC general disorders and administration site conditions was the more commonly reported for patients treated with TCZ, mainly due to the high occurrence of injection-related reactions [81]. A higher risk of developing a neurological disorder was observed for ADA. It is widely known that TNFi are mostly associated with central nervous system disorders [28]. A significant association was observed for UST and the onset of hepatobiliary disorders. In many studies, liver injury and increased transaminase values in patients treated with UST were uncommon [82]. Nevertheless, one case of severe transaminases elevation was reported in the literature [83]. Furthermore, the higher probability of developing respiratory complications with IFX, especially dyspnea, could be due to the infusion-related reactions [20]. Conversely, TCZ seems to be safe in patients with interstitial lung disease [84], but a review describes a possible onset of non-infectious pulmonary disorders, including a cough, as shown in our results [85].

Focusing on SAEs, our patients in treatment with TCZ, ADA, IFX, and ETN experienced serious neutropenia mostly associated with lymphocytosis as anti-TNFα drugs cause a dysregulation of TNFα ligands, including T cells. Furthermore, neutropenia with TCZ is undoubtedly due to the inhibition of the biologic influence of IL-6 on the recruitment of neutrophils into peripheral blood [86,87]. The onset of uveitis could be considered an extra-articular manifestation of inflammatory arthritis. Our cases of uveitis were mostly related to the use of ETN, as reported in another study [88]. Immunomodulation therapy in patients with autoimmune disease is one of the most common risk factors for experiencing PML. A case report described an occurrence of PML in a patient treated with ADA as happened in our findings [89]. It is well known that anti-TNFα therapy could lead to a myocardial infarction particularly in patients that are non-responders after six months of treatment supporting the idea that inflammation plays a crucial role in the onset of myocardial ischemia, although in some studies ADA had a decreased risk of incidence compared to csDMARDs [90,91]. Cancer risk during anti-TNFα treatment in patients with inflammatory arthritis may not be not increased as reported in several literature data, even if a relative risk of 1.3 was observed for breast cancer [92,93,94].

### Strengths and Limitations

Our study has some strengths and limitations. The main strength is that we conducted a multiregional pharmacovigilance study able to widen knowledge on biologics in a real-world setting. Indeed, our data confirmed the importance of pharmacovigilance activities to detect unknown AEs in clinical practice as demonstrated in several studies [18,33].

Our results regarded only two Italian regions, accounting for about 11.5% of the entire Italian population. The choice of a specific bDMARD on the index date could be related to the history of patients and it might depend on different regional directives that regulate the prescription of biological drugs and their biosimilars. Nevertheless, clinical characteristics associated with the geographical area should not modify the estimated safety profile of biologics. Moreover, our sample size may reflect clinical rheumatology practice especially compared to the limitation of pre-marketing randomized clinical trials, whose data may not directly be extended to “real-life” conditions. Furthermore, we performed a statistical analysis for confounding factors that may have influenced AE occurrence or primary/secondary failures and we took several factors into account, such as disease duration, smoking, concomitant therapies and comorbidities.

The rate of discontinuations increased over time especially for the initiating therapy with tsDMARDs. Additionally, the rate of patients lost to follow-up could create biases in patients stopping biological agents because of sustained remission or transferring to other rheumatologic units. The limited duration of the study (three years) did not allow for the detection of longer-term AEs, such as cancer, and it would be interesting to observe the effectiveness and safety of biological treatment after 5–10 years. For this reason, further investigations are necessary to obtain a better view of biological therapies in inflammatory arthritis.

## 5. Conclusions

Our study reports data for more than 1000 patients affected by inflammatory arthritis and treated with biologics in a real-world setting during a three-year study period. Our results reflect current clinical practice in the use of biologics in the rheumatologic area. Moreover, our findings reveal a better safety profile for SEC compared to ETN and a lower probability of experiencing therapeutic failures with GOL, SEC and TCZ. The reported AEs, generally mild to moderate and mostly related to infections and skin disorders, were all known. SAEs mostly regarded blood and lymphatic disorders, specifically neutropenia and lymphopenia and only two cases of malignancies were identified. These results suggest that some safety concerns still remain undetected, especially those related to long-term treatment. A switch/swap was observed in slightly more than 20% of patients, and treatment discontinuation or patients lost to follow-up were less than 6%. In the next few years, further studies are required to include other rheumatologic centers and new biologics currently in the marketing phase for inflammatory arthritis and to increase information on both effectiveness and safety profiles of bDMARDs.

## Figures and Tables

**Table 1:** Characteristics of patients treated with biologic disease-modifying antirheumatic drugs (bDMARDs) during the period 2016–2018.

Characteristic	Patients without AEs/Failures	Patients with AEs	*p*-Value ^1,2^	Patients with Failures	*p*-Value ^2,3^
	*n*= 785	*n* = 101		*n* = 269	
**Sex, *n* (%)**					
Females	476 (60.6)	72 (71.3)	**0.038**	205 (76.2)	**<0.001**
Males	309 (39.4)	29 (28.7)		64 (23.8)	
F/M ratio	1.5	2.5		3.2	
Median age (Q1–Q3)	57.0 (48.0–64.7)	57.0 (49.0–66.5)	0.593	57 (48.1–65.0)	0.696
Median age at diagnosis (Q1–Q3)	48.0 (39.0–56.0)	49.0 (34.5–55.5)	0.632	47.0 (38.0–55.0)	0.163
**Diagnosis, *n* (%)**					
Rheumatoid arthritis	343 (43.7)	48 (47.5)	0.466	140 (52.0)	**0.018**
Psoriatic arthritis	306 (39.0)	30 (29.7)	0.070	106 (39.4)	0.902
Anchylosing spondylitis	122 (15.5)	20 (19.8)	0.272	22 (8.2)	**0.002**
Non-radiographic axial spondyloarthritis	14 (1.8)	3 (3.0)	0.413	1 (0.4)	-
**Smoking, *n* (%)**					
Smoker	173 (22.0)	23 (22.8)	0.312	65 (24.2)	0.264
Ex-smoker	85 (10.8)	6 (5.9)		37 (13.8)	
Non-smoker	527 (67.1)	72 (71.3)		167 (62.1)	
CH index, median (Q1–Q3)	1.0 (0.0–1.0)	1.0 (0.0–1.0)	0.069	1.0 (0.0–1.0)	0.123
Comorbidities, median (Q1–Q3)	0.0 (0.0–1.0)	1.0 (0.0–2.5)	**<0.001**	0.0 (0.0–1.0)	0.901
**Comorbidities, *n* (%)**					
Hypertensive disease	155 (19.7)	27 (26.7)	0.102	46 (17.1)	0.341
Disorders of the thyroid gland	59 (7.5)	21 (20.8)	**<0.001**	22 (8.2)	0.725
Diabetes mellitus	61 (7.8)	10 (9.9)	0.458	18 (6.7)	0.562
Pure hypercholesterolemia	53 (6.8)	4 (4.0)	0.282	22 (8.2)	0.432
Fibromyalgia	38 (4.8)	9 (8.9)	0.086	27 (10.0)	**0.002**
Osteoporosis ^4^	36 (4.6)	11 (10.9)	**0.008**	10 (3.7)	0.547
Heart disease ^5^	31 (3.9)	6 (5.9)	0.346	14 (5.2)	0.379
Chronic lower respiratory diseases	26 (3.3)	14 (13.9)	**<0.001**	8 (3.0)	0.786
Noninfective enteritis and colitis	23 (2.9)	6 (5.9)	0.109	5 (1.9)	0.346
Mixed anxiety and depressive disorder	15 (1.9)	7 (6.9)	**0.002**	12 (4.5)	**0.022**
Viral hepatitis	18 (2.3)	5 (5.0)	0.114	6 (2.2)	0.953
Diseases of the eye and adnexa	11 (1.4)	5 (5.0)	**0.012**	4 (1.5)	0.918
Diseases of esophagus, stomach and duodenum	10 (1.3)	4 (4.0)	**0.042**	10 (3.7)	**0.011**
Uveitis	9 (1.1)	4 (4.0)	**0.027**	2 (0.7)	0.575
Concomitant non-biologics, median (Q1–Q3)	0.0 (0.0–1.0)	0.0 (0.0–1.0)	0.505	0.0 (0.0–1.0)	**0.028**
**csDMARDs, *n* (%)**					
Ciclosporin	4 (0.5)	0 (0.0)	-	6 (2.2)	**0.012**
Methotrexate	252 (32.1)	37 (36.6)	0.360	95 (35.3)	0.333
Leflunomide	19 (2.4)	0 (0.0)	-	13 (4.8)	**0.047**
Hydroxychloroquine	20 (2.5)	1 (1.0)	-	8 (3.0)	0.708
Sulfasalazine	12 (1.5)	3 (3.0)	0.239	3 (1.1)	0.621
Corticosteroid	33 (4.2)	8 (7.9)	0.094	21 (7.8)	**0.021**
Median age at biologic index date (Q1–Q3)	53.0 (44.0–60.0)	53.0 (43.0–61.0)	0.546	53.0 (44.0–61.0)	0.475

Abbreviations: AEs = adverse events, Q1 *=* first quartile, Q3 = third quartile; CH = Charlson, and csDMARDs = conventional synthetic disease-modifying antirheumatic drugs. ^1^ Patients without AEs or failures versus patients with AEs (Pearson’s chi-squared test or Mann–Whitney U test). ^2^ Bold indicates the statistically significant *p*-values. ^3^ Patients without AEs or failures versus patients with failures (Pearson’s chi-squared test or Mann–Whitney U test). ^4^ Osteoporosis with and without pathological fracture. ^5^ Including ischemia and other forms of heart disease.

**Table 2:** Safety risk profile and treatment failures of bDMARDs compared to Etanercept.

bDMARD	AEs vs ETN ^1^			Primary/Secondary Failures vs ETN ^1^		
	*N* of Cases (%)	OR Crude (95% CI)	OR Adjusted ^2^ (95% CI)	*N* of Cases (%)	OR Crude (95% CI)	OR Adjusted ^3^ (95% CI)
**ABT**	13 (13.7)	1.61 (0.79–3.29)	1.20 (0.53–2.74)	18 (18.9)	0.68 (0.38–1.21)	0.66 (0.37–1.19)
**ADA**	22 (8.4)	1.06 (0.58–1.91)	1.17 (0.63–2.18)	74 (28.4)	1.08 (0.75–1.56)	1.18 (0.81–1.73)
**CZP**	3 (9.7)	1.13 (0.32–4.04)	1.51 (0.41–5.62)	7 (22.6)	0.80 (0.33–1.96)	0.75 (0.30–1.90)
**GOL**	8 (8.8)	0.87 (0.38–1.99)	0.62 (0.24–1.58)	10 (11.0)	0.33 (0.16–0.67)	0.31 (0.15–0.65)
**IFX**	13 (12.1)	1.72 (0.84–3.52)	1.76 (0.80–3.90)	34 (31.8)	1.37 (0.84–2.22)	1.45 (0.88–2.39)
**RTX**	0 (0.0)	-	-	1 (9.1)	0.24 (0.03–1.91)	0.19 (0.02–1.55)
**SEC**	1 (1.3)	0.12 (0.02–0.86)	0.10 (0.01–0.82)	8 (10.2)	0.28 (0.13–0.61)	0.31 (0.14–0.67)
**TCZ**	11 (11.0)	1.10 (0.53–2.32)	0.99 (0.43–2.26)	10 (10.0)	0.31 (0.15–0.62)	0.34 (0.17–0.70)
**UST**	1 (2.9)	0.40 (0.05–3.07)	0.64 (0.08–5.16)	14 (40.0)	1.69 (0.82–3.49)	1.47 (0.67–3.26)

Abbreviations: ABT = abatacept, ADA = adalimumab, CI = confidence interval, CZP = certolizumab pegol, ETN = etanercept, GOL = golimumab, IFX = infliximab, OR = odds ratio, RTX = rituximab, SEC = secukinumab, TCZ = tocilizumab, and UST = ustekinumab. ^1^ All values are in reference to Etanercept, taking into account that etanercept was the first drug authorized for RA and was the most widely used drug in our sample (patients in treatment with ETN, *n* = 342, 29.6%: patients with AEs, *n* = 28, 8.2% and patients with primary/secondary failures, *n* = 92, 26.9%).^2^ Adjusted for sex, number of comorbidities, osteoporosis, disorders of the thyroid gland, gastrointestinal disease, eye disease, uveitis, mixed anxiety-depressive disorder, and respiratory disease. **^3^** Adjusted for sex, fibromyalgia, mixed anxiety-depressive disorder, gastrointestinal disease, and number of concomitant non-biologic therapy.

**Table 3:** Switches/swaps between bDMARDs related to therapeutic failures or AEs.

Switch/Swap to											
**Switch/swap from**		**ABT**	**ADA**	**CZP**	**ETN**	**GOL**	**IFX**	**RTX**	**SEC**	**TCZ**	**UST**
	**ABT**		1 (2)			1				13	
	**ADA**	15 (1)		5 (1)	17 (1)	8 (2)	1	1	7 (2)	9	1
	**ANA**									1	
	**CZP**				1				2 (1)	2	
	**ETN**	11	30 (6)	3		8 (2)	1		11	11 (1)	4
	**GOL**		2	(1)	4				3	1	
	**IFX**	2 (3)	3 (4)	2	11 (1)	9 (3)			1 (1)	3	
	**RTX**	1									
	**SEC**		3	1	2	1					
	**TCZ**	4 (4)	1		2 (2)	1					
	**UST**			1	1	1			6		

Switches/swaps related to therapeutic failures while switches/swap related to AEs were reported in brackets. The cells with grey background indicate a practically impossible switch/swap from/to the same biologic drug. Abbreviations: ABT = abatacept, ADA = adalimumab, CZP = certolizumab pegol, ETN = etanercept, GOL = golimumab, IFX = infliximab, RTX = rituximab, SEC = secukinumab, TCZ = tocilizumab, and UST = ustekinumab. No switches to anakinra (not reported).

**Table 4:** Adverse event distribution by bDMARDs according to MedDRA^®^ System Organ Class classification.

Adverse Event, *n* (%)	ABT	ADA	CZP	ETN	GOL	IFX	SEC	TCZ	UST	Total
Infections and infestations	9 (9.5) ^1^	9 (3.4)	2 (6.5)	10 (2.9)	10 (11.0) ^2^	2 (1.9)	3 (3.8)	2 (2.0)		47 (4.1)
Musculoskeletal and connective tissue disorders	4 (4.2)	6 (2.3)	3 (9.7)	11 (3.2)	5 (5.5)		2 (2.6)	1 (1.0)	6 (17.1) ^2^	38 (3.3)
Skin and subcutaneous tissue disorders	6 (6.3)	1 (0.4) ^1^	1 (3.2)	10 (2.9)	3 (3.3)	8 (7.5) ^1^	1 (1.3)	3 (3.0)	2 (5.7)	35 (3.0)
General disorders and administration site conditions		3 (1.1)		11 (3.2)		5 (4.7)		5 (5.0) ^1^		24 (2.1)
Respiratory, thoracic and mediastinal disorders	1 (1.1)	2 (0.8)		3 (0.9)		4 (3.7) ^1^		4 (4.0) ^1^		14 (1.2)
Blood and lymphatic system disorders		3 (1.1)		4 (1.2)		2 (1.9)		4 (4.0) ^1^		13 (1.1)
Investigations		7 (2.7) ^2^				2 (1.9)				9 (0.8)
Nervous system disorders		5 (1.9) ^1^		1 (0.3)		2 (1.9)				8 (0.7)
Eye disorders	1 (1.1)			5 (1.5) ^1^		1 (0.9)				7 (0.6)
Gastrointestinal disorders	1 (1.1)	1 (0.4)			1 (1.1)	1 (0.9)		1 (1.0)		5 (0.4)
Hepatobiliary disorders								1 (1.0)	2 (5.7) ^1^	4 (0.3)
Reproductive system and breast disorders				2 (0.6)						2 (0.2)
Neoplasms benign, malignant and unspecified		1 (0.4)			1 (1.1)					2 (0.2)
Vascular disorders			1 (3.2)	1 (0.3)						2 (0.2)
Ear and labyrinth disorders	1 (1.1)	1 (0.4)								2 (0.2)
Cardiac disorders		1 (0.4)		1 (0.3)						2 (0.2)
Injury, poisoning and procedural complications						1 (0.9)				1 (0.1)
Psychiatric disorders		1 (0.4)								1 (0.1)

Abbreviations: ABT = abatacept, ADA = adalimumab, CZP = certolizumab pegol, ETN = etanercept, GOL = golimumab, IFX = infliximab, SEC = secukinumab, TCZ = tocilizumab, and UST = ustekinumab. For anakinra only one case of hypertransaminasemia was registered (not reported). Percentages were calculated on the total of patients treated with each reference bDMARD. *p*-values were calculated comparing patients with AEs treated with each bDMARD versus patients with AEs treated with other bDMARDs (Chi-square test or Fisher exact test). ^1^ *p*-value ≤ 0.05. ^2^ *p*-value ≤ 0.001.

**Table 5:** Serious adverse event distribution by bDMARDs according to MedDRA^®^ classification.

Serious Adverse Event, *n* (%)	ABT	ADA	CZP	ETN	GOL	IFX	SEC	TCZ	Total
**Infections and infestations**	**3 (33.3)**	**5 (55.6)**	**2 (100)**	2 **(20.0)**	2 **(20.0)**	**2 (100)**	1 **(33.3)**	**2 (100)**	**19 (40.4)**
Bronchitis	1 (100)		1 (100)						2 (28.6)
Eye infection					1 (100)				1 (100)
Herpes simplex		1 (100)				1 (100)			2 (100)
Herpes zoster						1 (100)			1 (20.0)
Lung infection		1 (100)							1 (100)
Measles	1 (100)								1 (100)
Oral fungal infection				1 (100)					1 (100)
Oral infection					1 (100)				1 (100)
Osteomyelitis								1 (100)	1 (100)
Papilloma viral infection				1 (100)					1 (100)
Paronychia								1 (100)	1 (100)
Pneumonia			1 (100)						1 (100)
Progressive multifocal leukoencephalopathy		1 (100)							1 (100)
Rhinitis	1 (100)								1 (100)
Tooth abscess		2 (100)					1 (100)		3 (100)
**Blood and lymphatic system disorders**		**3 (100)**		**4 (100)**		**2 (100)**		**4 (100)**	**13 (100)**
Leukopenia								1 (100)	1 (100)
Lymphocytosis		1 (100)		2 (100)		1 (100)		1 (100)	5 (100)
Neutropenia		2 (100)		2 (100)		1 (100)		2 (100)	7 (100)
**Respiratory, thoracic and mediastinal disorders**	**1 (100)**	**2 (100)**				**3 (75.0)**			**6 (42.9)**
Acute respiratory failure		1 (100)							1 (100)
Chronic obstructive pulmonary disease	1 (100)								1 (100)
Cough						1 (100)			1 (50.0)
Dyspnea						1 (50.0)			1 (50.0)
Pulmonary mass		1 (100)							1 (100)
Tonsillar hypertrophy						1 (100)			1 (100)
**Eye disorders**				**4 (80.0)**		**1 (100)**			**5 (71.4)**
Glaucoma				1 (100)					1 (100)
Uveitis				3 (75.0)		1 (100)			4 (80.0)
**Nervous system disorders**		**1 (20.0)**		**1 (100)**					**2 (25.0)**
Demyelination				1 (100)					1 (100)
Syncope		1 (100)							1 (100)
**Neoplasms benign, malignant and unspecified**		**1 (100)**			**1 (100)**				**2 (100)**
Basal cell carcinoma					1 (100)				1 (100)
Breast cancer		1 (100)							1 (100)
**Cardiac disorders**		**1 (100)**		**1 (100)**					**2 (100)**
Atrial fibrillation				1 (100)					1 (100)
Myocardial infarction		1 (100)							1 (100)
**Skin and subcutaneous tissue disorders**	**2 (33.3)**								**2 (5.7)**
Skin exfoliation	1 (100)								1 (100)
Umbilical hemorrhage	1 (100)								1 (100)
**General disorders and administration site conditions**						**1 (20.0)**		**1 (20.0)**	**2 (8.3)**
Influenza-like illness								1 (100)	1 (100)
Pyrexia						1 (100)			1 (50.0)
**Gastrointestinal disorders**					**1 (100)**				**1 (20.0)**
Crohn’s disease					1 (100)				1 (100)
**Reproductive system and breast disorders**				**1 (50.0)**					**1 (50.0)**
Cervix disorder				1 (100)					1 (100)

Abbreviations: ABT = abatacept, ADA = adalimumab, CZP = certolizumab pegol, ETN = etanercept, GOL = golimumab, IFX = infliximab, SEC = secukinumab, and TCZ = tocilizumab. No serious adverse event to anakinra and ustekinumab (not reported). Bold indicates values for MedDRA^®^ System Organ Class classification.

## Supplementary Materials

The following are available online at https://www.mdpi.com/2077-0383/9/4/1227/s1, Table S1: Adverse event distribution by bDMARDs according to MedDRA^®^ System Organ Class and Preferred Term classification.
