# Supplementary material for: Safety Profile of Biologics Used in Rheumatology: An Italian Prospective Pharmacovigilance Study
Source: J Clin Med. 2020 Apr 24;9(4):1227. doi: 10.3390/jcm9041227 (PMC7230621; doi:10.3390/jcm9041227)
Supplement: Supplementary file 1 [file jcm-09-01227-s001.pdf]

Supplementary Materials

Table S1. Adverse event distribution by bDMARDs according to MedDRA® System Organ Class and Preferred Term classification

Adverse event, *n* (%)	ABT	ADA	CZP	ETN	GOL	IFX	SEC	TCZ	UST	Total
**Infections and infestations**	**9 (9.5)**	**9 (3.4)**	**2 (6.5)**	**10 (2.9)**	**10 (11.0)**	**2 (1.9)**	**3 (3.8)**	**2 (2.0)**		**47 (4.1)**
Bronchitis	1 (1.1)	1 (0.4)	1 (3.2)		3 (3.3)		1 (1.3)			7 (0.6)
Cystitis		1 (0.4)								1 (0.1)
Ear infection				1 (0.3)						1 (0.1)
Eye infection					1 (1.1)					1 (0.1)
Herpes simplex		1 (0.4)				1 (0.9)				2 (0.2)
Herpes zoster	1 (1.1)			2 (0.6)		1 (0.9)	1 (1.3)			5 (0.4)
Infection				1 (0.3)						1 (0.1)
Influenza	3 (3.2)	1 (0.4)		3 (0.9)	2 (2.2)					9 (0.8)
Localized infection					1 (1.1)					1 (0.1)
Lung infection		1 (0.4)								1 (0.1)
Measles	1 (1.1)									1 (0.1)
Molluscum contagiosum	1 (1.1)									1 (0.1)
Oral candidiasis		1 (0.4)								1 (0.1)
Oral fungal infection				1 (0.3)						1 (0.1)
Oral herpes	1 (1.1)									1 (0.1)
Oral infection					1 (1.1)					1 (0.1)
Osteomyelitis								1 (1.0)		1 (0.1)
Otitis media					1 (1.1)					1 (0.1)
Papilloma viral infection				1 (0.3)						1 (0.1)
Paronychia								1 (1.0)		1 (0.1)
Pneumonia			1 (3.2)							1 (0.1)
Progressive multifocal leukoencephalopathy		1 (0.4)								1 (0.1)
Rhinitis	1 (1.1)									1 (0.1)
Tonsillitis				1 (0.3)						1 (0.1)
Tooth abscess		2 (0.8)					1 (1.3)			3 (0.3)
Tooth infection					1 (1.1)					1 (0.1)
**Musculoskeletal and connective tissue disorders**	**4 (4.2)**	**6 (2.3)**	**3 (9.7)**	**11 (3.2)**	**5 (5.5)**		**2 (2.6)**	**1 (1.0)**	**6 (17.1)**	**38 (3.3)**
Arthralgia	1 (1.1)	1 (0.4)	1 (3.2)							3 (0.3)
Arthritis	1 (1.1)	4 (1.5)		9 (0.9)	3 (3.3)		2 (2.6)	1 (1.0)	3 (8.6)	23 (2.0)
Dactylitis				1 (0.3)					1 (2.9)	2 (0.2)
Enthesopathy					1 (1.1)					1 (0.1)
Flank pain	1 (1.1)									1 (0.1)
Joint swelling	1 (1.1)									1 (0.1)
Pain in extremity		1 (0.4)								1 (0.1)
Psoriatic arthropathy			1 (3.2)						2 (5.7)	3 (0.3)
Rheumatoid arthritis			1 (3.2)	1 (0.3)						2 (0.2)
Sacroiliitis					1 (1.1)					1 (0.1)
**Skin and subcutaneous tissue disorders**	**6 (6.3)**	**1 (0.4)**	**1 (3.2)**	**10 (2.9)**	**3 (3.3)**	**8 (7.5)**	**1 (1.3)**	**3 (3.0)**	**2 (5.7)**	**35 (3.0)**
Butterfly rash						1 (0.9)				1 (0.1)
Eczema				1 (0.3)						1 (0.1)
Hyperhidrosis						1 (0.9)				1 (0.1)
Mechanical urticaria				1 (0.3)						1 (0.1)
Nail disorder					1 (1.1)					1 (0.1)
Nodular rash	1 (1.1)									1 (0.1)
Palmar erythema					1 (1.1)					1 (0.1)
Pruritus			1 (3.2)	2 (0.6)		2 (1.9)				5 (0.4)
Psoriasis				2 (0.6)	1 (1.1)		1 (1.3)			4 (0.3)
Rash	2 (2.1)	1 (0.4)		2 (0.6)		2 (1.9)		2 (2.0)	1 (2.9)	10 (0.9)
Rash pruritic	1 (1.1)			2 (0.6)					1 (2.9)	4 (0.3)
Skin exfoliation	1 (1.1)									1 (0.1)
Umbilical hemorrhage	1 (1.1)									1 (0.1)
Urticaria						2 (1.9)		1 (1.0)		3 (0.3)
**General disorders and administration site conditions**		**3 (1.1)**		**11 (3.2)**		**5 (4.7)**		**5 (5.0)**		**24 (2.1)**
Administration site pruritus								1 (1.0)		1 (0.1)

Asthenia		1 (0.4)				1 (0.9)			2 (0.2)
Chest pain		1 (0.4)		2 (0.6)					3 (0.3)
Condition aggravated				4 (1.2)			1 (1.0)		5 (0.4)
Influenza like illness							1 (1.0)		1 (0.1)
Injection site hypersensitivity						1 (0.9)			1 (0.1)
Injection site pain				1 (0.3)			1 (1.0)		2 (0.2)
Injection site urticaria		1 (0.4)		3 (0.9)		1 (0.9)			5 (0.4)
Malaise						1 (0.9)			1 (0.1)
Pyrexia				1 (0.3)		1 (0.9)			2 (0.2)
Swelling							1 (1.0)		1 (0.1)
**Respiratory, thoracic and mediastinal disorders**	**1 (1.1)**	**2 (0.8)**		**3 (0.9)**		**4 (3.7)**	**4 (4.0)**		**14 (1.2)**
Acute respiratory failure		1 (0.4)							1 (0.1)
Asthma				1 (0.3)					1 (0.1)
Bronchospasm				1 (0.3)					1 (0.1)
Chronic obstructive pulmonary disease	1 (1.1)								1 (0.1)
Cough						1 (0.9)	1 (1.0)		2 (0.2)
Dysphonia							1 (1.0)		1 (0.1)
Dyspnoea				1 (0.3)		2 (1.9)			3 (0.3)
Laryngeal pain							1 (1.0)		1 (0.1)
Oropharyngeal pain							1 (1.0)		1 (0.1)
Pulmonary mass		1 (0.4)							1 (0.1)
Tonsillar hypertrophy						1 (0.9)			1 (0.1)
**Blood and lymphatic system disorders**		**3 (1.1)**		**4 (1.2)**		**2 (1.9)**	**4 (4.0)**		**13 (1.1)**
Leukopenia							1 (1.0)		1 (0.1)
Lymphocytosis		1 (0.4)		2 (0.6)		1 (0.9)	1 (1.0)		5 (0.4)
Neutropenia		2 (0.8)		2 (0.6)		1 (0.9)	2 (2.0)		7 (0.6)
**Investigations**		**7 (2.7)**				**2 (1.9)**			**9 (0.8)**
Antinuclear antibody positive		1 (0.4)				1 (0.9)			2 (0.2)
DNA antibody positive						1 (0.9)			1 (0.1)
Histone antibody positive		1 (0.4)							1 (0.1)
Protein C increased		1 (0.4)							1 (0.1)
Red blood cell sedimentation rate increased		3 (1.1)							3 (0.3)
Rheumatoid factor increased		1 (0.4)							1 (0.1)
**Nervous system disorders**		**5 (1.9)**		**1 (0.3)**		**2 (1.9)**			**8 (0.7)**
Crawling sensation		1 (0.4)							1 (0.1)
Demyelination				1 (0.3)					1 (0.1)
Dizziness		2 (0.8)				1 (0.9)			3 (0.3
Headache						1 (0.9)			1 (0.1)
Paresthesia		1 (0.4)							1 (0.1)
Syncope		1 (0.4)							1 (0.1)
**Eye disorders**	**1 (1.1)**			**5 (1.5)**		**1 (0.9)**			**7 (0.6)**
Blurred vision	1 (1.1)								1 (0.1)
Glaucoma				1 (0.3)					1 (0.1)
Uveitis				4 (1.2)		1 (0.9)			5 (0.4)
**Gastrointestinal disorders**	**1 (1.1)**	**1 (0.4)**			**1 (1.1)**	**1 (0.9)**	**1 (1.0)**		**5 (0.4)**
Abdominal distension	1 (1.1)								1 (0.1)
Crohn’s disease					1 (1.1)				1 (0.1)
Nausea		1 (0.4)				1 (0.9)			2 (0.2)
Oral pain							1 (1.0)		1 (0.1)
**Hepatobiliary disorders**							**1 (1.0)**	**2 (5.7)**	**4 (0.3)**
Hepatic steatosis								1 (2.9)	1 (0.1)
Hypertransaminasemia							1 (1.0)	1 (2.9)	3 (0.3)
**Reproductive system and breast disorders**				**2 (0.6)**					**2 (0.2)**
Benign prostatic hyperplasia				1 (0.3)					1 (0.1)
Cervix disorder				1 (0.3)					1 (0.1)
**Neoplasms benign, malignant and unspecified**		**1 (0.4)**			**1 (1.1)**				**2 (0.2)**
Basal cell carcinoma					1 (1.1)				1 (0.1)
Breast cancer		1 (0.4)							1 (0.1)
**Vascular disorders**			**1 (3.2)**	**1 (0.3)**					**2 (0.2)**

Flushing			1 (3.2)	1 (0.3)						2 (0.2)
**Ear and labyrinth disorders**	**1 (1.1)**	**1 (0.4)**								**2 (0.2)**
Tinnitus	1 (1.1)									1 (0.1)
Vertigo		1 (0.4)								1 (0.1)
**Cardiac disorders**		**1 (0.4)**		**1 (0.3)**						**2 (0.2)**
Atrial fibrillation				1 (0.3)						1 (0.1)
Myocardial infarction		1 (0.4)								1 (0.1)
**Injury, poisoning and procedural complications**						**1 (0.9)**				**1 (0.1)**
Infusion related reaction						1 (1.9)				1 (0.1)
**Psychiatric disorders**		**1 (0.4)**								**1 (0.1)**
Confusional state		1 (0.4)								1 (0.1)

Abbreviations: ABT = abatacept, ADA = adalimumab, CZP = certolizumab pegol, ETN = etanercept, GOL = golimumab, IFX = infliximab, SEC = secukinumab, TCZ = tocilizumab, and UST = ustekinumab. For nakinra only one case of hypertransaminasemia was registered (not reported). Percentages were calculated on the total of patients treated with each reference bDMARD. Bold indicates values for MedDRA® System Organ Class classification.
